# Magnetic Resonance Imaging as a Biomarker for Renal Cell Carcinoma

**DOI:** 10.1155/2015/648495

**Published:** 2015-11-01

**Authors:** Yan Wu, Young Suk Kwon, Mina Labib, David J. Foran, Eric A. Singer

**Affiliations:** ^1^Center for Biomedical Imaging & Informatics, Rutgers Cancer Institute of New Jersey and Rutgers Robert Wood Johnson Medical School, New Brunswick, NJ 08903, USA; ^2^Department of Radiology, Rutgers Cancer Institute of New Jersey and Rutgers Robert Wood Johnson Medical School, New Brunswick, NJ 08903, USA; ^3^Section of Urologic Oncology, Rutgers Cancer Institute of New Jersey and Rutgers Robert Wood Johnson Medical School, New Brunswick, NJ 08903, USA

## Abstract

As the most common neoplasm arising from the kidney, renal cell carcinoma (RCC) continues to have a significant impact on global health. Conventional cross-sectional imaging has always served an important role in the staging of RCC. However, with recent advances in imaging techniques and postprocessing analysis, magnetic resonance imaging (MRI) now has the capability to function as a diagnostic, therapeutic, and prognostic biomarker for RCC. For this narrative literature review, a PubMed search was conducted to collect the most relevant and impactful studies from our perspectives as urologic oncologists, radiologists, and computational imaging specialists. We seek to cover advanced MR imaging and image analysis techniques that may improve the management of patients with small renal mass or metastatic renal cell carcinoma.

## 1. Introduction

RCC accounts for approximately 90% of all renal malignancies and continues to pose a global health risk with the highest rates observed in Czech Republic and North America [1]. In the United States, 61,560 new cases and 14,080 deaths are expected in 2015 alone [2]. The steady rise in incidence over the past few decades is likely associated with the expanding use of imaging tests in medical practice and recent advances in imaging technology in the diagnosis of localized cancer. However, this does not fully explain the increased incidence rate in nonwestern countries, where many patients still present with advanced disease at the time of initial diagnosis [3]. Our review will discuss the challenges clinicians face in the diagnosis and management of patients with small renal masses as well as metastatic renal cell carcinoma. We describe the development of advanced magnetic resonance imaging and image analysis techniques that can potentially ameliorate some of the challenges in these areas of renal oncology.

## 2. Challenge of the Small Renal Mass

**Figure 1:**
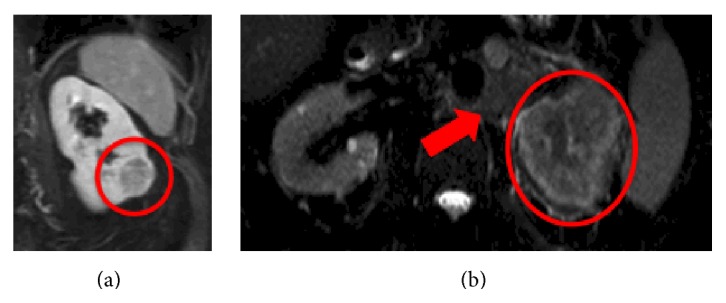
Conventional MRI provides anatomic but not physiologic information about kidney tumors. (a) 3 cm exophytic renal mass is imaged with conventional MRI that can only provide information about the size of a renal mass and its enhancement after administration of gadolinium-based contrast agent. Based on its size, there is a 30% likelihood that it is benign. Percutaneous core needle biopsy determined that it was a renal cell carcinoma. (b) 7 cm endophytic renal mass with para-aortic lymphadenopathy indicated by the red arrow.

Traditionally, computed tomography (CT) has been considered the gold standard for imaging of renal masses since the 1990s and the utility of multiphasic multidetector CT has been described in differentiating clear-cell RCC from other histologic subtypes [4]. Magnetic resonance imaging (MRI) has also been used with at least comparable or even better sensitivity [5]. Willatt et al. suggest that a previously considered indeterminate lesion on CT imaging can be better evaluated with MRI for tumor characteristics consistent with malignancy [6]. This is important as 30–40% of small renal masses (SRM) ≤ 4 cm and up to 20% of masses ≤7 cm are benign [7]. A conventional MRI image of a patient with a SRM is shown in Figure 1(a).

Despite improvements in imaging technology, the management of small renal masses (SRM) remains challenging, especially when trying to differentiate benign from malignant tumors. Although the standard of care for localized SRM remains surgical resection, with surveillance and ablation offered to carefully selected patients, percutaneous renal mass biopsy (RMB) is gaining popularity. It can provide valuable information on the pathological, molecular, and genetic characteristics of the SRM and is used to classify SRM for optimal clinical management [8–12]. Richard et al. demonstrated that RMB is safe and reliable and can decrease unnecessary treatments in favor of surveillance in a long-term study of 13 years [13].

However, RMB is not without its limitations. First, sampling error can occur which results in a nondiagnostic biopsy. In the review of 2,474 RMB results, positive predictive value (PPV) and negative predictive value (NPV) for the diagnosis of malignancy were 97.5% and 82.0%, respectively; overall sensitivity and specificity were 92.1% and 89.7%, respectively [14].

Despite the high rate of diagnostic accuracy, the rate of nondiagnostic biopsy remains substantial, which has limited the widespread use of RMB [14]. Leveridge et al. reported that the rate of nondiagnostic biopsy at his institution was approximately 20%. Interestingly, the diagnostic rate on repeat biopsy was similar to that on initial biopsy, arguing against the distinct intrinsic properties of tumors that resulted in the nondiagnostic findings at first [15]. A more recent single-institution study of 529 patients demonstrated improved diagnostic accuracy with nondiagnostic biopsy rate of 10% [13]. Multivariate analysis has shown that larger tumor size and exophytic location were associated with obtaining a diagnostic biopsy [13]. While both of these studies support the role of repeat biopsy as a feasible and useful next step in characterizing the indeterminate tumor [13, 15], it should also be noted that each RMB comes with a risk of complications.

Although RMB procedure is generally considered safe with minimal long-term consequences, awareness of patient characteristics and potential complications is important. In a single-center study, the vast majority of complications were related to bleeding and only required conservative management [13]. In the review of contemporary series, there has not been a case of tumor seeding of the biopsy tract, the most detrimental complication of RMB, since the introduction of coaxial techniques with guides or cannulas [16]. Nonetheless, risks should be carefully assessed in a patient with comorbidities and suboptimal information in order to further minimize the incidence of any adverse events [17].

Despite the improved diagnostic accuracy and safety of RMB, there is an inherent complexity of tumor characteristics that cannot be easily delineated with a single biopsy. This challenge stems from intratumoral heterogeneity [18]. In this situation, both favorable and unfavorable gene profiles are expressed in different regions of the same tumor. Moreover, different regions of the tumor may have different mutations in the very same genes [19]. While tumor heterogeneity is more common in metastatic cancer where disseminated cancer cells exhibit more diverse genomic compositions, a recent report by Ball et al. demonstrated that there is considerable nuclear degree heterogeneity in SRM as well [10]. Because of tumor heterogeneity, a biopsy may not adequately sample the most aggressive area of the tumor or sufficiently gauge the extent of genomic deviations within it, making therapeutic decision-making more challenging.

Lastly, histologic characteristics may not reliably separate RCCs from benign tumors. For example, it is known that chromophobe RCC is not easily distinguishable from renal oncocytomas on core needle biopsy alone. Given this similarity, efforts have been made to differentiate tumors at the genetic level by looking for either rearrangements or translocations [20]. Taken together, an alternative diagnostic approach to renal mass biopsy should be investigated.

## 3. Challenge of Advanced Renal Cell Carcinoma

At the opposite end of disease spectrum, there have been significant shifts in the treatment of patients with advanced RCC. A MRI image of a patient with advanced RCC is shown in Figure 1(b). Previously, high dose interleukin-2 (HD IL-2) was essentially the only efficacious treatment of metastatic RCC (mRCC). This is a toxic but potentially curative therapy for a subset of carefully selected patients with metastatic clear-cell RCC [21, 22] who have an excellent performance status with minimal medical comorbidities. However, starting in 2006 with the approval of the vascular endothelial growth factor receptor (VEGFR) antagonists sunitinib and sorafenib, cytokine therapy diminished while targeted therapies (TT) grew rapidly in number and utilization [23–28]. A recent population-based study by Allard et al. further illustrates the national trends of HD IL-2 and TT utilization in the United States from 2004 to 2012 [29]. The authors found that the administration of HD IL-2 has been limited to a minority of patients with excellent performance status because of the associated acute toxicities. However, recent increases in HD IL-2 utilization from its nadir in 2008 also suggest the inability of TT to deliver the desired therapeutic outcome.

The efficacy of TT has also been limited, as complete responses remain extremely rare [30]. For example, the response rate for all mRCC patients treated with sunitinib was 16.4% in a recent community-based cohort [31]. Of these, only 3 patients (2.2%) had a complete response and 19 patients (14.2%) had a partial response [31]. Furthermore, treatment-related toxicity with contemporary TT remains considerable as 45 patients (39.5%) had their dose reduced and 26 patients (22.8%) discontinued sunitinib entirely.

Therefore, reflexive first-line use of TT should be approached with caution and HD IL-2 should still be considered for appropriately selected patients. Unfortunately, we do not yet have the ability to reliably predict which patients will respond best to TT or HD IL-2.

As the role of imaging in RCC has expanded beyond diagnosis and cancer staging to potentially include prognosis, treatment selection, and response to therapy, it is hoped that advanced imaging methods that evaluate pathologic angiogenesis in tumor can further contribute to individually tailoring RCC therapy while minimizing unnecessary procedures and treatment toxicities [32]. In particular, advanced MRI techniques may help pave the way for a new chapter in RCC management.

## 4. Clinical Applications of Advanced Imaging and Image Analysis Techniques

Using radiology images as biomarkers has been approached from various perspectives in renal oncology. Some researchers have used novel imaging methods during data acquisition whereas others applied image analysis techniques as part of postprocessing procedure. In the former category, perfusion MRI and diffusion MRI play important roles in tumor characterization, prediction, and early detection of therapeutic response. These functional imaging methods are promising, since they reflect physiologic information within the tumor in addition to anatomic information such as size. They may be particularly useful in the evaluation of novel therapies, such as antiangiogenic therapy or immunotherapy. In the latter category, radiomics analysis has begun to attract more attention. With the advent of image processing and machine learning techniques, pertinent information on texture, shape, and margin of the renal tumors can be extracted from anatomic or physiologic images. This information has the potential to assist urologists and oncologists in making optimal treatment decisions for their patients with SRM or metastatic RCC.

## 5. Perfusion MRI for Assessing RCC Histology and Predicting Response to Therapy

**Figure 2:**
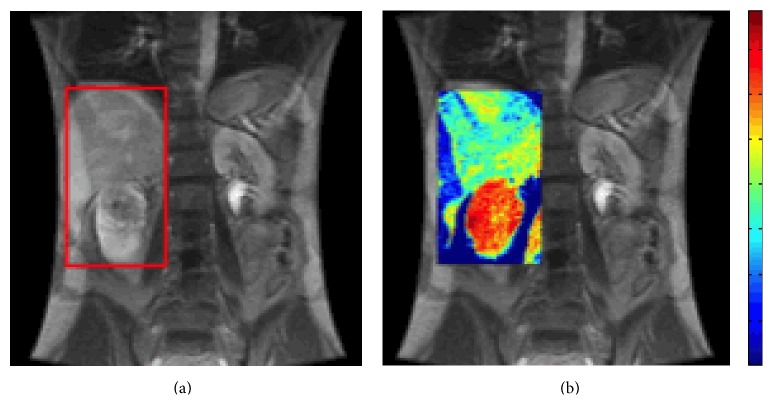
Perfusion MRI. T1-weighted MRI (a) and dynamic contrast-enhanced pMRI (b) of a renal mass: a series of 3D images were acquired. Each 3D image consists of 8 coronal slices with a 5-second acquisition time. Subsequently, 3D perfusion parametric map showing the microcirculation of the renal mass was generated. Red color in the tumor is indicative of a high level of perfusion. Surgical pathology revealed clear-cell RCC, Fuhrman grade 4, with sarcomatoid features.

There are three different types of perfusion MRI (pMRI) techniques: dynamic contrast-enhanced (DCE), dynamic susceptibility contrast (DSC), and arterial spin labeling (ASL). In DCE and DSC pMRI, signal intensity on postcontrast MRI images is changed after the intravenous injection of gadolinium-based contrast agent (GBCA). Based on postprocessing models, the change in signal intensity can be used to measure perfusion parameters. Particularly for DCE pMRI, a T1-weighted gradient echo sequence is used after the administration of GBCA contrast agent at a medium rate of 1–3 mL/s. The raw MR images with hyperintense lesions are postprocessed based on the pharmacokinetic model, and perfusion parametric maps (Ktrans) are generated. The result from a DCE pMRI study is shown in Figure 2. For DSC pMRI, on the other hand, a T2-weighted fast sequence (echo planar imaging or fast spin echo sequence) is used after the injection of GBCA (at a faster rate >4 mL/s). Here, the raw MR images with hypointense lesions are postprocessed according to the indicator dilution theory, where perfusion parametric maps consist of blood flow and blood volume.

ASL perfusion does not require the administration of GBCA. Instead, water protons in the blood are used as the tracer and are labeled by inversion pulses before they are imaged using a fast proton density weighted imaging sequence (echo planar imaging or fast spin echo sequence). Therefore, ASL perfusion can be used in patients with poor renal function who cannot tolerate GBCA at the cost of relatively low signal-to-noise ratio (SNR) and relatively long acquisition time.

Because conventional imaging methods have not been able to consistently distinguish benign from malignant renal masses, pMRI has been used to differentiate the histology of renal masses in some preliminary studies. Lanzman et al. conducted an ASL pMRI study on 34 patients and found that mean and peak perfusion levels within the tumor area in a single slice can be used to distinguish different histopathologic subtypes of renal masses: oncocytomas demonstrated higher perfusion levels than RCCs and papillary RCCs exhibited lower perfusion levels than other RCC subtypes [33]. Similarly, Chandarana et al. obtained Ktrans of the entire tumor from 24 patients using modified kinetics models to discriminate chromophobe RCC from other renal lesions [34]. In addition to evaluating different RCC subtypes, a study by Palmowski et al. reported that DCE pMRI can also be used to estimate the morphologic grading of RCC and hence assess adverse oncologic features. This was accomplished by calculating perfusion levels in the entire tumor, as well as in the most vascularized part of the tumor, and then correlating those values with the surgical pathology [35].

In addition to its improved diagnostic capabilities, perhaps the most exciting application of pMRI is its potential to help oncologists manage patients with mRCC who are being treated with TT. Flaherty et al. reported that a significant decrease in Ktrans was observed in a small cohort of patients who achieved partial response with sorafenib therapy [36]. Moreover, the percent decline in Ktrans, as well as baseline Ktrans, was significantly associated with progression-free survival and tumor shrinkage [36]. Similarly, Hahn et al. studied 44 evaluable subjects with mRCC treated with sorafenib. They reported that high baseline Ktrans was associated with longer progression-free survival but that the reduction in Ktrans after four weeks of TT was not predictive of progression-free survival when adjusted for the dose [37].

In a cohort of 17 patients who underwent treatment with PTK787/ZK 222584, a VEGFR TKI, de Bazelaire et al. demonstrated a significant decrease in ASL blood flow in patients with stable disease or partial response when compared to those with progressive disease [38]. Furthermore, the change in ASL blood flow measured at 1 month was correlated with the change in tumor size measured at 4 months and time to progression. Taken together, these findings indicate that decreased ASL blood flow could be an early predictor of clinical response to antiangiogenic TT.

While several studies have demonstrated that pMRI is promising in the management of patients with RCC, they have some common limitations. The statistical power of these studies was weakened by the small number of total patients or patients with specific tumors [34–36, 38]. Histologic diagnosis might be less accurate if it was obtained from core needle biopsy rather than surgical pathology [33]. Most importantly, no standardized pMRI acquisition method has been established, making it difficult to compare results obtained at different medical facilities.

Reaching consensus on a standard imaging protocol is an important task but remains challenging. Effective pMRI acquisition requires cooperation from radiologists, medical physicists, and experienced MRI technicians as technical pitfalls that degrade imaging quality are common [39]. Measures of standardization for an abdominal pMRI study include imaging sequence, respiratory maneuver (e.g., free breathing or breath hold, as well as related acquisition with or without respiratory trigger), imaging parameters (e.g., flip angle, injection rate of contrast agent, and range of the tumor area to image), and postprocessing methods.

For example, different ranges of the tumor area can be selected for imaging. Some researchers prefer to image the whole tumor at the cost of reduced temporal resolution although it takes a longer acquisition time for a 3D image. As a result, the accuracy of estimating perfusion parameters from a dynamic series of images will be compromised due to the reduced temporal resolution. To counter this problem, others may prefer to acquire a single slice image at higher temporal resolution through the area thought to represent the most aggressive part of the tumor. However, the area of interest might not be captured due to respiratory motion, and the ability to investigate tumor heterogeneity will be limited by the small range of acquired images.

Since the efficacy of pMRI has not been proven with prospective studies, the incorporation of pMRI into routine clinical practice is not yet feasible. Our institution is currently enrolling subjects on a pilot study using DCE pMRI as diagnostic, therapeutic, and prognostic biomarker for patients with organ-confined renal masses or mRCC (NCT02526511).

## 6. Diffusion-Weighted Imaging for Assessing RCC Histology and Predicting Response to Therapy

**Figure 3:**
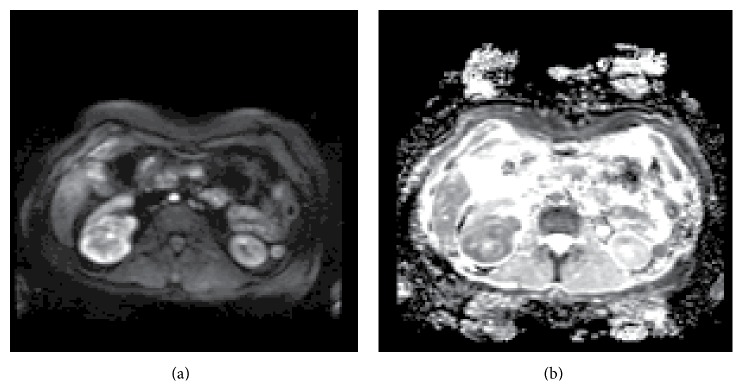
Diffusion-weighted MRI. The DWI (a) and ADC (b) images of the same patient were acquired in the axial direction using a *b* value of 800 s/mm^2^. This high-grade clear-cell RCC appears hyperintense on DWI, showing restricted diffusion (a). This was confirmed by hypointensity on the ADC map (b).

Diffusion, or Brownian motion, of tissues can be measured using specially designed diffusion-weighted imaging (DWI) sequences. The result of a DWI study is shown in Figure 3. The signal intensity in DWI is determined by an operator specific factor *b* and a tissue specific factor, apparent diffusion coefficient (ADC). The *b* factor indicates how much diffusion weighting is applied. A small *b* value is associated with high signal intensity while a large *b* value indicates an increased level of tissue contrast. Therefore, a small *b* value is good for detecting a lesion with higher signal intensity, whereas a large *b* value is better for tumor characterization with higher tissue contrast. Most lesions with restricted diffusion appear hyperintense in DWI and hypointense in the ADC map.

DWI has been used to differentiate various subgroups of renal masses. In a cohort of 42 patients, Sandrasegaran et al. found that ADC of cystic renal cancers was lower than that of benign cysts, and ADC of malignant lesions was lower than that of benign lesions; high-grade RCC was more likely to have lower ADC when compared to low-grade RCC. However, there was no significant difference in ADC levels between clear-cell and non-clear-cell RCCs [40].

Other investigators, in contrast, have been able to differentiate clear-cell RCC from non-clear-cell RCC, although conflicting ADC levels have been reported. For instance, Paudyal et al. reported a significantly lower ADC value in clear-cell RCC compared to non-clear-cell RCC in a cohort of 47 patients [41]. However, Wang et al. observed a significantly higher ADC value in clear-cell RCC in a cohort of 85 patients [42]. A possible explanation for this discrepancy between the two studies could be the exclusion of necrotic tumor components of RCC in the analysis in the former study. Because the necrotic portion of RCC is associated with high ADC levels, the ADC level would be significantly decreased when the analysis is limited to a nonnecrotic portion of the tumor. It should also be noted that a significant difference in ADC between papillary RCC and chromophobe RCC was only observed when a large *b* value (800 sec/mm^2^) was used instead of a small *b* value (500 sec/mm^2^), demonstrating that large *b* values have better capability in tumor characterization [42].

Similarly, ADC levels were found to be useful in differentiating benign lesions from RCC. Tanaka et al. suggested that clear-cell RCC and minimal fat angiomyolipoma (MFAML) could be distinguished based on their marked differences in ADC values, which otherwise would be challenging with conventional imaging [43]. Taouli et al. conducted a comprehensive study on 64 patients using DWI and contrast-enhanced MRI [44]. Mean ADC was able to distinguish RCC from benign lesions as well as papillary RCC from nonpapillary RCCs. Differentiation from contrast-enhanced MRI was more accurate when compared to that from ADC in terms of area under the curve, sensitivity, and specificity. However, the combination of DWI and contrast-enhanced MRI had the best specificity [44], suggesting the role of combined imaging in differentiating renal lesions with improved accuracy.

In addition to ADC, intravoxel incoherent motion (IVIM) imaging parameters were also used in recent years to discriminate renal tumor subtypes based on the biexponential model. Chandarana et al. demonstrated the differentiation of renal tumor subtypes in 26 patients using perfusion fraction and tissue diffusivity, which were extracted from diffusion-weighted images acquired using 8 *b* values [45]. Perfusion fraction was also shown to have a good correlation with semiquantitative perfusion parameter AUC60, potentially enabling the assessment of tumor vascularity without the need for GBCA [45].

Although interesting results have been reported with this technique as shown in Table 1, previous studies of DWI have similar limitations to pMRI. The small sample size was a concern in some studies. They had either insufficient total number of patients or very few patients with specific histologic subtypes [40, 41, 45]. In other studies that characterized RCC subtypes, benign renal lesions were not included as a control group, which may limit the use of ADC values for differentiating benign lesions from malignant ones [41]. Secondly, pathologic diagnosis of some patients was missing in a few studies. Because some diagnoses of benign lesions were made from follow-up scans, the chances of misclassification may exist [44].

In addition, different *b* values were used, ranging from 500 to 1000 s/mm^2^. However, *b* values will influence the accuracy of ADC measurement. For the same patients, slightly different ADC values will be obtained depending on which *b* values are used [42]. Comparing ADC values obtained at different hospitals with various *b* values would be questionable, although a general trend could be observed. Therefore, it is important to establish a standard imaging protocol with consensus on *b* values in the future, as DWI has been more widely incorporated in abdomen MRI protocols due to its short scan time and potential clinical utility.

## 7. Multiparametric MRI for Assessing RCC Histology and Predicting Response to Therapy

It is also feasible to use multiparametric MRI, which consists of DWI and pMRI, for the characterization of renal lesions and the assessment of TT treatment response. Notohamiprodjo et al. used pMRI and DWI for histological differentiation [46]. Multiple DCE pMRI parameters were obtained based on a two-compartment exchange model. The DCE pMRI parameters, including plasma flow, plasma volume, permeability surface area product, and extravascular extracellular volume, were correlated with tumor oxygenation. ADC from DWI was moderately correlated with the extracellular volume but was not related to tumor oxygenation or perfusion. In comparison, DCE pMRI appeared superior to DWI for histological differentiation.

Desar et al. investigated early vascular changes after sunitinib was given to 10 patients with RCC [47]. At day 3 and day 10, the relative tumor blood volume and relative tumor blood flow were significantly decreased based on DSC pMRI, supporting their roles as potential early treatment response biomarkers. However, there was no significant decrease in mean Ktrans. DWI showed a significant increase in ADC, followed by a decrease, indicating a change in cellularity, edema, and necrosis [47]. Thus, use of multiparametric MRI could provide more comprehensive information for personalized therapies, combining the strengths of both DWI and pMRI.

## 8. Radiomics Analysis for Assessing RCC Histology and Predicting Response to Therapy

Radiomics is an emerging field and refers to the process of extracting data from radiology images and using those data to noninvasively predict tumor phenotype. Radiomics analysis can evaluate the extent of tumor heterogeneity and better characterize the tumor and potentially predict the subsequent treatment response. Quantitative imaging features that can be extracted through radiomics analysis include textural features, functional parameters, and features from multiparametric imaging [48].

Radiomics analysis has been used to study the histology of renal masses. In a cohort of 61 patients, Kierans et al. identified texture metrics of ADC maps, including first-order skewness and second-order cooccurrence matrix, as significant independent predictors of stage, but did not find mean ADC to be a predictive factor [49]. This finding suggests that radiomics demonstrates a strong predictive power in differentiating cancer stage. Gaing et al. used statistical measures of mean, standard deviation, skewness, and kurtosis of IVIM parametric maps to differentiate malignant from benign lesions as well as various subtypes of renal cancers in 44 patients. Out of 15 subtype pairs, 8 out of 9 pairs were differentiated using mean and histogram of perfusion fraction and tissue diffusivity; clear-cell RCC was distinguished from AML using standard deviation of tissue diffusivity; oncocytoma was separated from AML, clear-cell RCC, and papillary RCC using kurtosis of perfusion fraction [50].

Radiomics analysis has begun to show potential as a predictive imaging biomarker of response to TT in patients with metastatic RCC. For example, Smith et al. established MASS (Morphology, Attenuation, Size, and Structure) criteria to evaluate tumor response to sorafenib and sunitinib in a cohort of 84 patients [51]. The MASS criteria showed higher accuracy and interobserver reliability than conventional SACT, RECIST, or modified Choi criteria. Similarly, Goh et al. applied texture analysis on CT images of 39 patients with metastatic RCC who received TT [52]. Using tumor entropy and texture uniformity at different scales, responders and nonresponders had significantly different survival patterns on Kaplan-Meier curves, showing some evidence that tumor entropy and texture uniformity criteria could be an improvement to the standard response criteria (RECIST, Choi, and modified Choi criteria) in terms of distinguishing responders and nonresponders [52]. Texture uniformity was also an independent predictor of time to progression on Cox regression analysis.

## 9. Conclusions

Advanced MR imaging techniques (pMRI and DWI) and radiomics analysis have the potential to serve as diagnostic, therapeutic, and prognostic RCC biomarkers. The aforementioned imaging biomarkers will continue to improve our ability to provide optimal counseling, treatment recommendations, and monitoring for patients with organ-confined or advanced RCC. Additional clinical trials are needed to further explore and optimize the role of advanced MRI in RCC.

## Figures and Tables

**Table 1:** Selected review of the literature on diffusion-weighted MRI.

Authors	Year	Sample size	*b* values (s/mm^2^)	Main findings
Sandrasegaran et al. [40]	2010	59 lesions (i) 20 benign (ii) 17 ccRCCs, 5 pRCCs, and 1 chRCC (iii) 2 TCCs	0, 400, and 800	ADCs of malignant tumors are lower than those of benign tumors. ADCs of high-grade ccRCC are lower than those of low-grade ccRCC.

Paudyal et al. [41]	2010	47 lesions (i) 25 ccRCCs (ii) 6 pRCCs (iii) 1 chRCC (iv) 15 TCCs	0, 300, and 1000	Significant differences exist in ADCs between clear-cell RCCs and non-clear-cell RCCs, between RCCs and TCCs, and between positive and negative metastatic lesions.

Wang et al. [42]	2010	85 lesions (i) 49 ccRCCs (ii) 22 pRCCs (iii) 14 chRCCs	0, 500, and 800	Mean ADC (acquired with 800 sec/mm^2^) allows differentiation of RCC subtypes with 95.9% sensitivity and 94.4% specificity, whereas mean ADCs (acquired with 500 sec/mm^2^) cannot differentiate between pRCC and chRCC.

Tanaka et al. [43]	2011	41 lesions (i) 36 ccRCCs (ii) 5 MFAMLs	0, 800	Clear-cell RCC exhibits more heterogeneous signal on ADC map than MFAML.

Taouli et al. [44]	2009	109 lesions (i) 81 benign (ii) 28 RCCs	0, 400, and 800	Mean ADC is able to differentiate RCC from benign lesions and papillary RCC from nonpapillary RCCs. DCE MRI was more accurate than ADC, but the combination of the two had the best specificity.

Chandarana et al. [45]	2012	26 lesions (i) 14 ccRCCs (ii) 5 chRCCs (iii) 3 cystic RCCs	0, 50, 100, 150, 250, 400, 600, and 800	The combination of perfusion fraction and tissue diffusivity can diagnose pRCC and cystic RCC with 100% accuracy and ccRCC and chRCC with 86.5% accuracy.

Notohamiprodjo et al. [46]	2013	18 lesions (i) 14 ccRCCs (ii) 4 pRCCs	0, 500	ADC shows moderate correlation with the extracellular volume but is not related to tumor oxygenation or perfusion.

Desar et al. [47]	2011	10 lesions	50, 300, and 600	A significant increase at day 3, followed by a decrease at day 10 in ADC, after sunitinib is applied to patients with RCC, indicating a change in cellularity, edema, and necrosis.

ccRCC: clear-cell renal cell carcinoma; pRCC: papillary renal cell carcinoma; chRCC: chromophobe renal cell carcinoma; TCC: transitional cell carcinoma; MFAML: minimal fat angiomyolipoma.
